# Endothelial glycocalyx shedding in the acute respiratory distress syndrome after flu syndrome

**DOI:** 10.1186/s40560-020-00488-7

**Published:** 2020-09-21

**Authors:** Maira Nilson Benatti, Alexandre Todorovic Fabro, Carlos Henrique Miranda

**Affiliations:** 1 Division of Emergency Medicine, Department of Internal Medicine, Ribeirão Preto School of Medicine, São Paulo University, Rua Bernardino de Campos, 1000, Ribeirão Preto, São Paulo, 14020-670 Brazil; 2 Department of Pathology and Legal Medicine, Ribeirão Preto School of Medicine, São Paulo University, Ribeirão Preto, São Paulo, Brazil

**Keywords:** Acute respiratory distress syndrome, Endothelial glycocalyx, Hyaluronic, Influenza virus

## Abstract

**Background:**

Scientific evidence indicates that endothelial glycocalyx (EG) shedding contributes to the pathophysiological installation of acute respiratory distress syndrome (ARDS) after bacterial sepsis. The aim was to evaluate the EG shedding in ARDS installation after flu syndrome.

**Methods:**

This cross-sectional study included patients with flu syndrome during the influenza outbreak divided into two groups: patients with and without ARDS. Healthy subjects without flu syndrome were included in a control group. We measured EG damage biomarkers (hyaluronan, syndecan-1) and endothelial cell injury biomarker (soluble thrombomodulin) during the first medical evaluation. Histological assessment of the perimeter of the hyaline membrane and the number of neutrophils infiltrated in the alveolar septum was performed in patients who died.

**Results:**

ARDS group had 30 patients (44 ± 16 years old, 57% men), the non-ARDS group had 36 patients (39 ± 17 years old, 42% men), and the control group had 35 individuals (44 ± 9 years old, 51% men). Hyaluronan levels were significantly higher in the ARDS group than the two groups [31 ng/ml (interquartile range-IQR 12–56) vs. 5 ng/ml (IQR 3–10) vs. 5 ng/ml (IQR 2–8); *p* < 0.0001]. Hyaluronan levels above 19 ng/ml in patients with flu syndrome were associated with a significant increase in 28-day mortality rate: relative risk (RR): 6.95; (95% confidence interval 1.88–25.67); *p* = 0.0017. A positive correlation was observed between hyaline membrane perimeter and soluble thrombomodulin levels (*r* = 0.89; *p* = 0.05) as well as between the number of neutrophils in the alveolar septum and hyaluronan levels (*r* = 0.89; *p* = 0.05).

**Conclusions:**

Evidence of EG shedding was found in ARDS established after flu syndrome.

## Background

Respiratory viruses are endemic worldwide and usually cause benign and self-limiting disease. Sometimes, they can reach the lower airway, especially the influenza virus, and trigger severe pneumonia associated with acute respiratory distress syndrome (ARDS) [1, 2]. At times, another respiratory virus can emerge and cause epidemics, such as recently observed with the SARS-CoV-2 virus [3]. The pathophysiological mechanism behind the establishment of this severe presentation is not precise.

Endothelial glycocalyx (EG) is a carbohydrate-rich gelatinous layer that covers the vessels internally [4, 5]. Damage to this structure leads to increased vascular permeability, adhesion, and neutrophil migration, which have a pivotal function in the installation of ARDS [6]. Recent investigations demonstrated the occurrence of EG shedding in the early phase of ARDS due to bacterial sepsis [7], but no research has evaluated the involvement of this structure in ARDS triggered after viral infections. This study aimed to investigate the occurrence of EG shedding through biomarkers of damage of this structure (hyaluronan and syndencan-1) and biomarker of endothelial cell injury (soluble thrombomodulin) and its participation in ARDS establishment after flu syndrome.

## Methods

### Study design

This cross-sectional, single-centered study included patients with flu syndrome admitted to the emergency department of our hospital (tertiary center) and another primary center, both located in the city of Ribeirão Preto. This primary health unit was chosen because it is part of the Sentinel Network, which is a national surveillance system of the Brazilian Health Ministry that monitors medical care for flu syndrome and circulation of the respiratory viruses. The study was approved by the Research Ethics Committee of our institution and followed the Declaration of Helsinki (approval number 3758/2016). The subjects or their relatives gave their written informed consent to participate in this research.

### Patients

Patients with flu syndrome, defined according to the World Health Organization (WHO) criteria with a documented episode of fever (temperature > 37.8 °C) associated with at least two of the following symptoms: chills, myalgia, dry cough, sore throat, prostration, and rhinorrhea during a period of the influenza virus outbreak from April 2016 to July 2016 and from April 2017 to July 2017.

Subsequently, the patients were divided into two distinct groups. The first was defined as flu syndrome with ARDS, those who met ARDS criteria according to the Berlin definition [8]: the presence of acute symptoms within the last 7 days, bilateral infiltrate on chest radiography, arterial hypoxemia with a ratio of a partial pressure of arterial oxygen (PaO_2_) to the fraction of inspired oxygen (FiO_2_) < 300 mmHg, and the absence of structural heart disease or evidence of acute heart failure. The second was defined as flu syndrome without the ARDS group, those who did not meet the above criteria. We decided to use the ARDS criteria instead of the severe acute respiratory syndrome (WHO) since the latter is less accurate because it considers any respiratory discomfort with arterial oxygen saturation below 93% in ambient air.

Patients were excluded who had a documented bacterial infection, presented a circulatory shock defined as systolic blood pressure < 90 mmHg or need for vasoactive drugs, suffered from some cancer, and had chronic or acute renal failure (creatinine > 2.0 mg/dl). For bacterial infection exclusion, we used a detailed clinical evaluation along with the time from illness onset to the medical evaluation. We excluded patients with respiratory symptoms longer than 10 days, fever longer than 7 days, purulent sputum, and lobar consolidation on chest radiography. Healthy individuals of both genders over 18 years of age with no respiratory symptoms or infection in the last week and not vaccinated for influenza in the previous 6 weeks were included in the control group. Demographic, clinical, and laboratory data were collected from medical records.

### Sample and laboratory tests

During the first medical assessment, a venous blood sample was collected in a heparinized tube. The blood was centrifuged at 3500×*g* for 15 min at 18 °C. The plasma was separated into 0.5 ml aliquots and stored in a freezer at − 70 °C. Biomarkers of EG shedding were measured through commercial ELISA kits (enzyme-linked immunosorbent assay): hyaluronan (R&D, Minneapolis, MN, USA), syndecan-1 (Abcam, Cambridge, MA, USA), and biomarker of endothelial cell injury: soluble thrombomodulin (R&D, Minneapolis, MN, USA); inflammatory cytokines were measured through commercial ELISA kits: tumor necrosis factor-alpha (TNF-α), interleukin-6 (IL-6), interleukin 1-beta (IL-1β) (both through R&D, Minneapolis, MN, USA), and quantification of immunoglobulin G and immunoglobulin M for influenza A (IBL America, Minneapolis, MN, USA). In patients with less than 5 days after the onset of symptoms, a nasopharyngeal swab was collected for direct immunofluorescence for respiratory viruses through the D3 Ultra DFA Respiratory Virus Screening & ID Kit (Quidel Corporation, San Diego, CA, USA). For the positive results on direct immunofluorescence, a real-time polymerase chain reaction (RT-PCR) for influenza A was conducted according to the US Centers for Disease Control and Prevention (CDC, Atlanta, GA, USA) protocol [9]. 

### Histological analysis

A pathologist reviewed the lung parenchyma stained by hematoxylin-eosin of the cases submitted to autopsy. The degree of damage to the alveolar-capillary membrane was determined through ten high-magnification photographs (×400) randomly taken using an optical microscope (Novel) coupled to the HD lite 1080p camera. The Image-Pro Plus software performed morphometric analyses. We measured the perimeter of the hyaline membrane divided by the perimeter determined by the alveolar septum. Likewise, the number of neutrophils was manually counted and divided by the perimeter determined by the alveolar septum. An average of these ten measurements was made for each patient.

### Statistical analysis

We used the Shapiro–Wilk test to evaluate the distribution of the continuous variable. Continuous variables with normal distribution were expressed as mean ± standard deviation, and other variables were expressed as the median and interquartile range (IQR). Student’s *t* test was used to assess the difference between two continuous variables with a normal distribution. The Mann-Whitney test was used to compare two continuous variables without normal distribution. Categorical variables were expressed as frequency or percentage. The chi-square test was used to compare two categorical variables. To compare the levels of biomarkers among the three groups, we used the ANOVA test with the Bonferroni post-test if the variables had a normal distribution, and the Kruskal-Wallis test was used, followed by the Dunn post-test when these variables did not have a normal distribution. We used the Spearman correlation coefficient to evaluate the correlation between two continuous variables. To determine the diagnosis and prognosis accuracy of each biomarker, we analyzed the area under the receiver operating characteristic (ROC) curve and its respective 95% confidence interval (95% CI). We used the Youden index to determine the best cutoff point for each biomarker. For survival analysis, Kaplan-Meier curves were constructed and compared using the log-rank test.

Initially, to estimate the sample size, we considered the need to include twelve patients in each group based in findings of a previous study [10] that observed a hyaluronan level of 10 ± 4 ng/ml in patients with ARDS and 2 ± 4 ng/ml in controls and a power of 80% and a significance level of 5%. This estimative was performed comparing the control (healthy individuals) with the ARDS patients. As we did not know the behavior of the hyaluronan levels in the intermediate group (flu syndrome without ARDS), in which we initially considered to have a middle level of hyaluronan, we decided to include at least double these initial number of patients (*n* = 24).

A two-tailed *p*-value less than or equal to 0.05 was considered statistically significant. Statistical analysis and graphs were performed using the GraphPad Prism software version 7.00 (California, USA) and the STATA software version 13 (College Station, TX, USA).

## Results

**Fig. 1:**
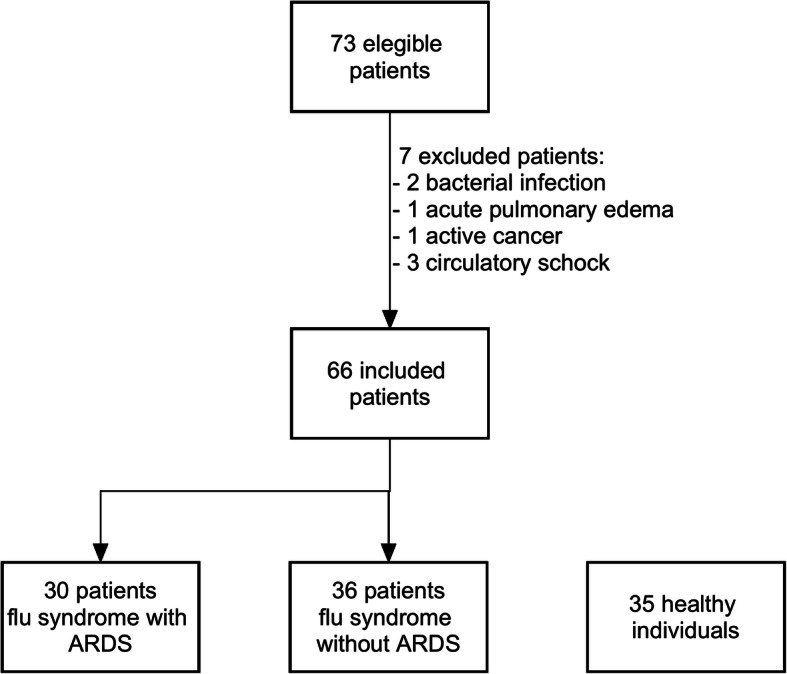
Diagram flow of the patients included in this investigation

A total of 101 individuals were included, 30 patients in flu syndrome with ARDS group, 36 patients in flu syndrome without ARDS group, and 35 individuals in the control group. The flow diagram of the patients included in this investigation is in Fig. 1. The demographic, clinical, and laboratory characteristics of these patients are provided in Table 1. Demographic characteristics were similar among the three groups. For the clinical presentation, a higher occurrence of dyspnea was observed in the flu syndrome with the ARDS group (97% vs. 39%, *p* < 0.0001). The duration of symptoms was slightly higher in the flu syndrome with the ARDS group (5 days vs. 4 days, *p* < 0.0001), apparently without clinical significance. There were no significant differences in the presence of epidemiological risk factors for severe clinical presentation after influenza infection.

**Table 1:** Characteristics of the patients included in this investigation.

Characteristics	Flu syndrome			***p value***
	With ARDS ***N*** = 30	Without ARDS ***N*** = 36	Control ***N*** = 35	
Demographic				
Age (years), mean ± sd	44 ± 16	39 ± 17	44 ± 9	0.280
Male gender, *n* (%)	17 (57)	15 (42)	18 (51)	0.700
Clinical presentation, *n* (%)				
Fever	28 (93)	32 (89)	–	0.680
Cough	26 (87)	28 (78)	–	0.670
Myalgia	18 (60)	27 (75)	–	0.280
Dyspnea	29 (97)	14 (39)	–	< 0.0001
Symptoms duration; days, median (IQR)	5 (4–7)	4 (2–5)	–	< 0.0001
Risk factors for severe presentation, *n* (%)				
Diabetes	05 (17)	02 (05)	–	0.430
Hypertension	07 (23)	03 (08)	–	0.170
Smoking	06 (20)	04 (11)	–	0.490
Pulmonary disease	7 (23)	5 (14)	–	0.350
Cardiac disease	2 (07)	2 (06)	–	1.000
Neurologic disease	2 (07)	0 (00)	–	0.200
Obesity	4 (13)	2 (06)	–	0.400
Pregnancy	0 (00)	0 (00)	–	1.000
Physical examination				
Heart rate; bpm, mean ± sd	104 ± 21	86 ± 15	–	0.0009
SBP; mmHg, mean ± sd	117 ± 21	119 ± 13	–	0.750
DBP; mmHg, mean ± sd	73 ± 15	74 ± 8	–	0.700
Laboratory test				
Creatinine; mg/dl, median (IQR)	1.0 (0.8–1.6)	0.9 (0.8–1.2)	–	0.200
Leukocytes; mm^3^, median (IQR)	6150 (3900–10100)	8350 (6300–10900)	–	0.050
Influenza A IgM positive, *n* (%)	8 (27)	10 (28)	–	0.930
Influenza A IgG positive, *n* (%)	30 (100)	35 (97)	–	1.000
Influenza A RT-PCR positive, *n*/*n*(%)	12/23*(52)	03/25*(12)	–	0.020
Influenza A RT-PCR or IgM positive, *n* (%)	17 (57)	13(36)	–	0.100
ARDS classification (Berlin criteria)				
Mild	6 (20)	–	–	
Moderate	7 (23)	–	–	
Severe	17 (57)	–	–	
PaO_2_/FiO_2_ ratio; mmHg, median (IQR)	88 (65–140)	–	–	
SOFA score, mean ± sd	7 ± 4	–	–	
Hospitalization rate, *n* (%)	30 (100)	06 (17)	–	< 0.0001
In-hospital mortality rate, *n* (%)	14 (47)	00 (00)	–	< 0.0001

*ARDS* acute respiratory distress syndrome, *sd* standard deviation, *IQR* interquartile range, *COPD* chronic obstructive pulmonary disease, *SBP* systolic blood pressure, *DBP* diastolic blood pressure, *IgM* immunoglobulin M, *IgG* immunoglobulin G, *RT-PCR* real-time polymerase chain reaction, *PaO*_*2*_ partial pressure of arterial oxygen, *FiO*_*2*_ fraction of inspired oxygen, *SOFA* sequential organ failure assessment * proportion between the positive results and number of collected samples

Laboratory confirmation of influenza A infection through the positive IgM or RT-PCR for influenza A virus was observed in 17 patients (57%) in the flu syndrome with ARDS group and in 13 patients (36%) in the flu syndrome without ARDS group, *p* = 0.1.

The results of the biomarker measurement are presented in Table 2. A higher level of hyaluronan in flu syndrome with the ARDS group [31 ng/ml (IQR 12–56)] was observed compared to the other two groups, flu syndrome without ARDS [5 ng/ml (IQR 3–10)], *p* = 0.0002, and control [5 ng/ml (IQR 2–8)], *p* < 0.0001. Plasma levels of thrombomodulin were higher in the flu syndrome with the ARDS group [1411 pg/ml (IQR 878–2237)] and flu syndrome without the ARDS group [1390 pg/ml (IQR 21–17460)] compared to the control group [219 pg/ml (IQR 21–601)], *p* = 0.0001. However, no significant difference was observed between the first two groups, *p* = 0.57. Plasma levels of syndecan-1 were higher in the flu syndrome with the ARDS group [28 ng/ml (IQR 21–601) 24–570] compared to flu syndrome without the ARDS group [24 ng/ml (IQR 24–25)], *p* = 0.009. However, no other differences were observed. The levels of the inflammatory cytokines were not different among the groups (*p* > 0.05 for all comparisons). In the analysis restricted to patients with laboratory-confirmed influenza A infection, the hyaluronan levels remain higher in the flu syndrome with the ARDS group [34 ng/ml (IQR 16–62)] than the other two groups; flu syndrome without ARDS [8 ng/ml (IQR 4–15)], *p* = 0.015, and control [5 ng/ml (IQR 2–8)], *p* = 0.006.

**Table 2:** Levels of the biomarkers of endothelial damage and cytokines in the three groups evaluated in this investigation

Biomarkers, median (IQR)	Flu syndrome			
	With ARDS *N* = 30	Without ARDS *N* = 36	Control *N* = 35	*p value*
Hyaluronan (ng/ml)	31 (12–56)	5 (3–10)	5 (2–8)	< 0.0001*
Thrombomodulin (pg/ml)	1411 (878–2237)	1390 (21–1746)	219 (21–601)	< 0.0001‡
Syndecan-1 (ng/ml)	28 (24–57)	24 (24–25)	24 (17–41)	0.011§
TNF-α (pg/ml)	15 (15–51)	15 (15–43)	23 (5–110)	0.850
IL-6 (pg/ml)	39 (12–129)	33 (6–83)	16 (6–41)	0.081
IL-1β (pg/ml)	4 (0.4–32)	0.4 (0.4–45)	4 (0.4–10)	0.788
	Positive RT-PCR or IgM for influenza A			
	With ARDS *N* = 17	Without ARDS *N* = 13	Control *N* = 35	
Hyaluronan (ng/ml)	34 (16–62)	8 (4–15)	5 (2–8)	0.001**
Thrombomodulin (pg/ml)	1374 (923–1980)	1339 (575–1888)	219 (21–601)	< 0.0001‡‡
Syndecan-1 (ng/ml)	24 (24–41)	24 (19–70)	24 (17–41)	0.427
TNF-α (pg/ml)	15(15–30)	15 (12–26)	23 (5–109)	0.745
IL-6 (pg/ml)	37 (1283)	52 (6–98)	16(6–41)	0.255
IL-1β (pg/ml)	4 (0.4–33)	4 (0.4–47)	4(0.4–10)	0.957

*ARDS* acute respiratory distress syndrome, *IQR* interquartile range

*with ARDS vs. without ARDS, *p* = 0.0002; with ARDS vs. control, *p* < 0.0001, without ARDS vs. control, *p* > 0.99

‡with ARDS vs. without ARDS, *p* = 0.57; with ARDS vs. control, *p* < 0.0001; without ARDS vs. control, *p* = 0.0001

§with ARDS vs. without ARDS, *p* = 0.009, with ARDS vs. control, *p* = 0.1536, without ARDS vs. control, *p* = 0.888

** with ARDS vs. without ARDS, *p* = 0.015; with ARDS vs. control, *p* = 0.006, without ARDS vs. control, *p* = 0.91

‡‡with ARDS vs. without ARDS, *p* = 0.99; with ARDS vs. control, *p* < 0.0001; without ARDS vs. control, *p* = 0.0008

**Fig. 2:**
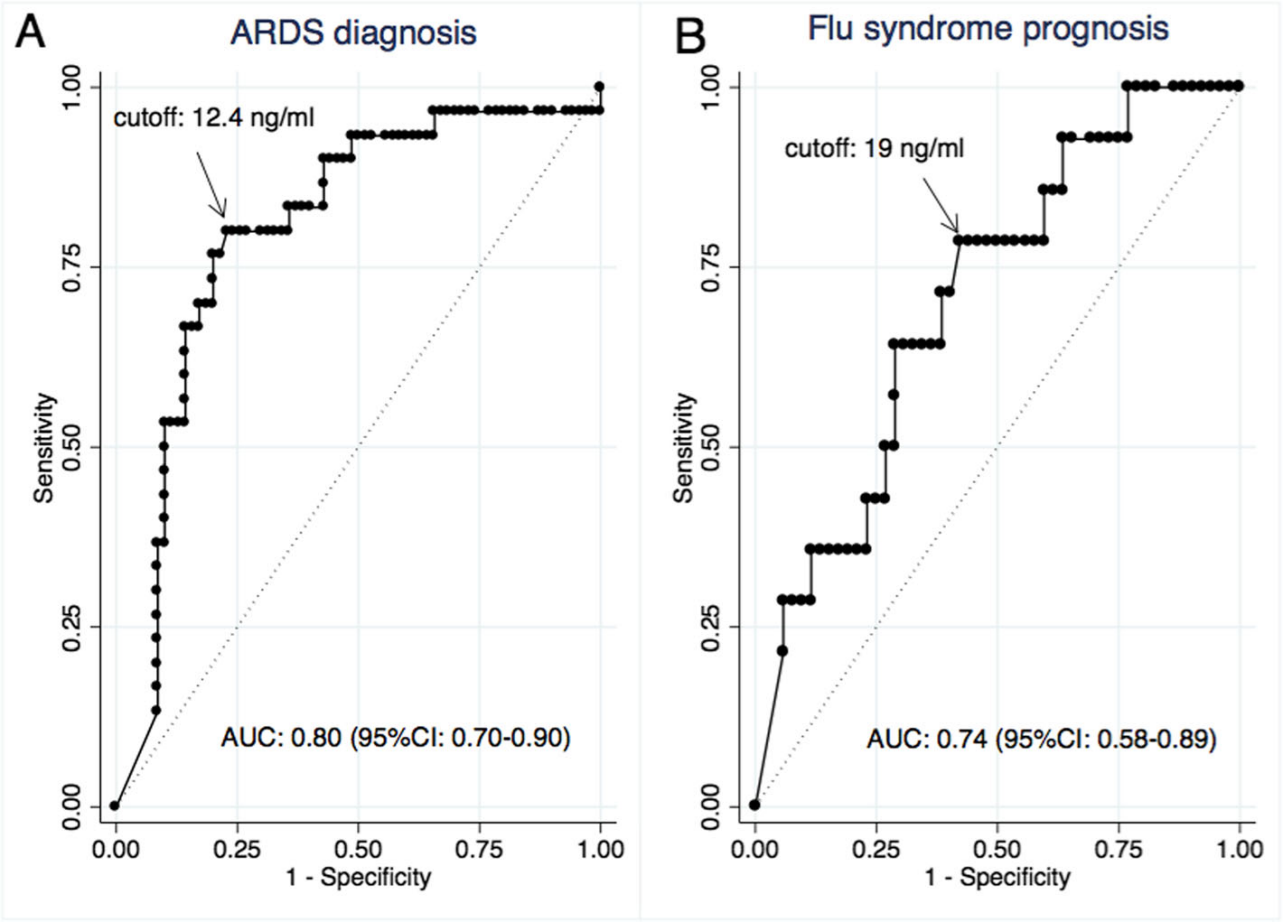
Receiver operating characteristic (ROC) curves of the hyaluronan levels and its particular cutoff point to determine acute respiratory distress syndrome (ARDS) diagnosis (**a**) and the prognosis (28-day mortality) in all patients with flu syndrome (**b**). AUC area under the curve, CI confidence interval

Through the ROC curve, the hyaluronan levels exhibited the better ARDS diagnosis accuracy with an area under the curve of 0.80 (95% CI 0.70–0.90), with the best cutoff point of 12.4 ng/ml (Fig. 2a) with a sensitivity of 80% (95% CI 61–92) and specificity of 79% (95% CI 68–88). Hyaluronan levels of > 12.4 ng/ml were associated with an ARDS diagnosis with an odds ratio (OR) of 15 (95% CI 5–43); *p* = 0.0001.

This paragraph contains the results of the analysis restricted to the flu syndrome with the ARDS group (*n* = 30). Thrombomodulin levels were higher in the severe [1417 pg/ml (IQR 1016–2242)] and the moderate group [1787 pg/ml (IQR 1405–2675)] than the mild group [528 pg/ml (IQR 931–12310)], *p* = 0.02. A significant difference was not observed in the syndecan-1 and hyaluronan levels according to the ARDS severity. There was no correlation between the levels of endothelial damage biomarkers and the PaO_2_/FiO_2_ ratio [hyaluronan (*r* = 0.01; *p* = 0.95), thrombomodulin (*r* = − 0.18, *p* = 0.33), and syndecan-1 (*r* = − 0.30, *p* = 0.12)]. The levels of these biomarkers were not different in surviving and non-surviving patients: hyaluronan [35 ng/ml (IQR 15–51) vs. 23 ng/ml (IQR 10–73)], *p* = 0.82; thrombomodulin [1390 pg/ml (IQR 699–1861) vs. 1539 pg/ml (IQR 1016–2353)], *p* = 0.50; and syndecan–1 [28 ng/ml (IQR 24–59) vs. 32 ng/ml (IQR 24–47)], *p* = 0.80.

**Fig. 3:**
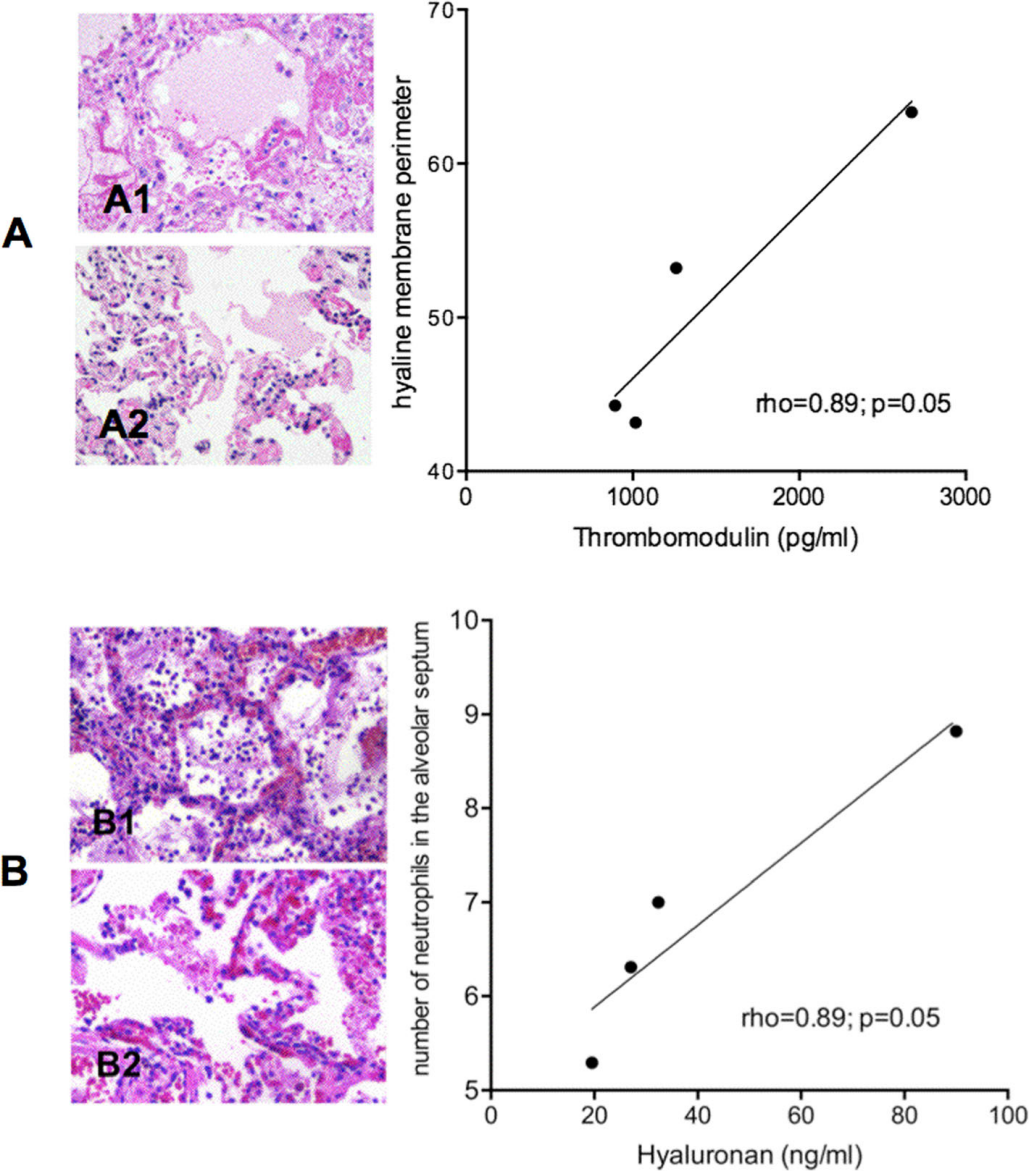
Scatter plot showing the correlation between the perimeter of the hyaline membrane and the thrombomodulin levels (**a**) and the number of neutrophils in the alveolar septum and the hyaluronan levels (**b**) in the four patients of the flu syndrome with acute respiratory distress syndrome (ARDS) group. A1) Hematoxylin-eosin (H&E) panel shows a hyaline membrane filling the entire alveolar space (×400). A2) H&E panel illustrates a hyaline membrane with a smaller dimension about the alveolar space (×400). B1) H&E panel displays an alveolar septum (×400) with a high number of neutrophils. B2) H&E panel B has an alveolar septum (×400) with a reduced number of neutrophils

We performed an autopsy and histological analysis for four of the twelve patients who died in the flu syndrome with the ARDS group. The demographic and clinical characteristics of these four patients are presented in Table 3. These patients were young with age ranging from 34 to 55 years old, three (75%) with severe ARDS, and one (25%) with moderate ARDS on admission. They died in the early course of hospitalization (1–9 days) while receiving protective mechanical ventilation with a low tidal volume. In this group, we observed a positive correlation between hyaline membrane perimeter and thrombomodulin levels (*r* = 0.89; *p* = 0.05) and between the number of neutrophils in the alveolar septum and hyaluronan levels (*r* = 0.89; *p* = 0.05) as presented in Fig. 3.

**Table 3:** A) Demographic and clinical characteristics of the patients with flu syndrome with ARDS who underwent autopsy. B) Correlation between biomarkers of endothelial damage and hyaline membrane perimeter and the number of neutrophils in the alveolar septum in these patients

**A) Characteristics of the patients who underwent autopsy**						
	Age (year)	Gender	Symptoms duration (days)	PaO_2_/FiO_2_ ratio on admission	Risk factors	Time for death after admission (days)
Patient 1	34	Female	4	65	Overweight	1
Patient 2	53	Male	6	85	Smoking	2
Patient 3	37	Female	4	57	Obesity	5
Patient 4	55	Female	6	110	Obesity, diabetes, hypertension	9
**B) Biomarkers and autopsy findings**						
Biomarker			Hyaline membrane perimeter		Number of neutrophils in alveolar septum	
			*r*	*p*-value	*r*	*p*-value
Hyaluronan			– 0.40	0.60	0.89	0.05
Thrombomodulin			0.89	0.05	– 0.30	0.69
Syndecan-1			0.87	0.13	– 0.17	0.83

*PaO*_*2*_ partial pressure of arterial oxygen, *FiO*_*2*_ fraction of inspired oxygen, *r* coefficient of correlation

The prognostic accuracy in determining the 28-day mortality among patients with flu syndrome for each biomarker was performed through the area under ROC-curve. The hyaluronan showed the best prognosis performance [AUC: 0.74 (95% CI 0.58–0.89) compared to the syndecan-1 [AUC: 0.57 (95% CI 0.37–0.78] and thrombomodulin [AUC: 0.57 (95% CI 0.38–0.78)]. The best cutoff point of the hyaluronan levels for prognosis determination was 19 ng/ml including all patients with flu syndrome (Fig. 2b).

**Fig. 4:**
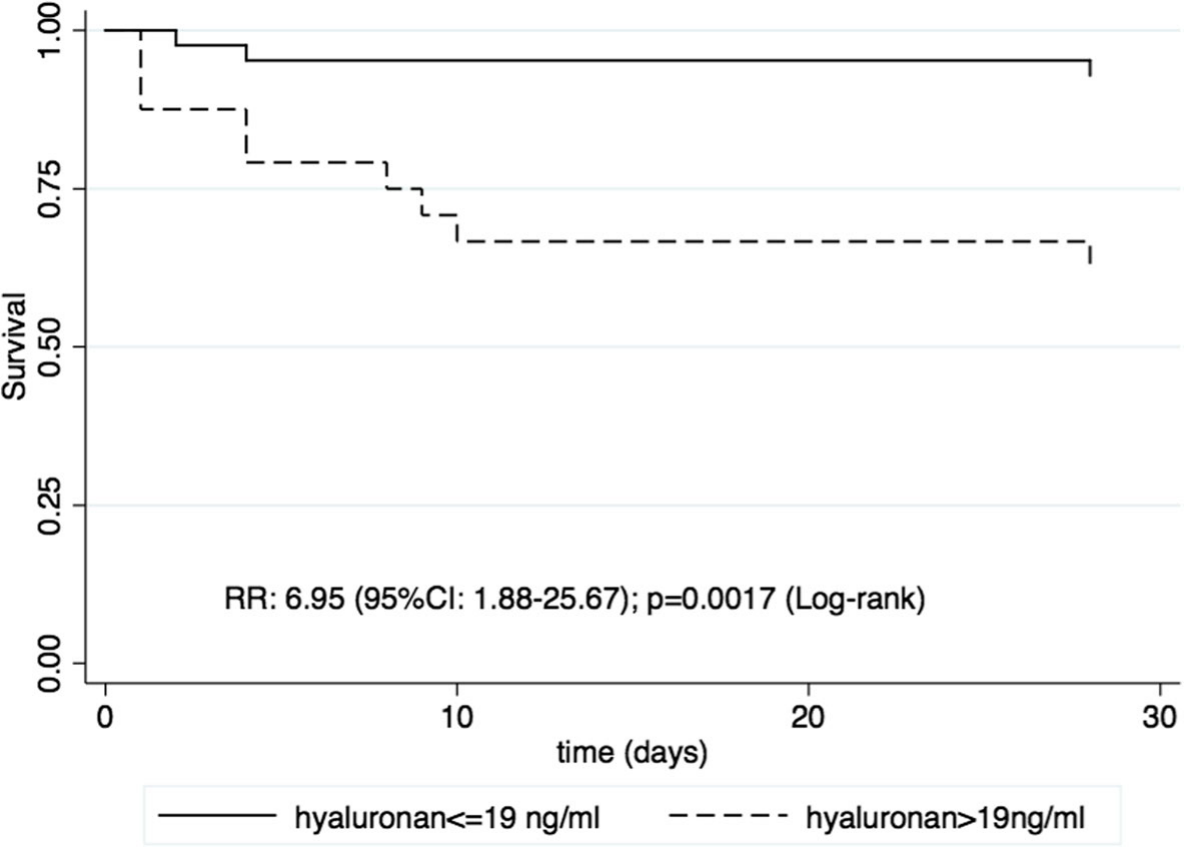
Kaplan-Meier curve showing the survival according to the hyaluronan levels in all patients with flu syndrome. RR relative risk, CI confidence interval

The result of the survival analysis is displayed in Fig. 4. The hyaluronan levels higher than 19 ng/ml in patients with flu syndrome were associated with a significant increase in 28-day mortality rate: relative risk (RR): 6.95 (95% CI 1.88–25.67), *p* = 0.0017.

## Discussion

Our findings demonstrated, for the first time, evidence that EG shedding happens during ARDS installation after flu syndrome. Furthermore, the hyaluronan levels were a diagnosis and prognosis biomarker to select patients with the worst clinical evolution.

In 2012, Schmidt et al. [7] were the first to propose, through an experimental investigation using intravital microscopy, that endotoxemia in mice rapidly induced pulmonary microvascular endothelial glycocalyx degradation via TNF-α, and it contributes to the neutrophil adhesion and lung injury installation. In this study, the authors found that heparanase inhibition prevented endotoxemia-associated EG shedding.

After this, several clinical investigations found an association between endothelial glycocalyx shedding and ARDS establishment. Murphy et al. [11] observed that syndecan-1 levels were associated with ARDS (*p* = 0.05), and these levels were independently predictive of mortality in multivariable logistic regression (odds ratio: 1.85 per log increase in syndecan-1 levels, *p* = 0.03). Esposito et al*.* [12] in 2017 analyzing 86 patients with ARDS showed a positive correlation between hyaluronan levels in serum (*p* = 0.04) and the bronchoalveolar lavage fluid (*p* = 0.03) with the lung injury score; moreover, the hyaluronan levels in bronchoalveolar lavage fluid were associated with the respiratory component of the Sequential Organ Failure Assessment Score (SOFA), *p* = 0.03. Lariviere et al. [13] also showed a critical role of the heparan sulfate in ARDS establishment. Recently, Schmidt et al. [14] demonstrated that early indices of urinary glycosaminoglycans fragmentation could predict acute kidney injury and in-hospital mortality in patients with septic shock or ARDS.

All these investigations evaluated the EG shedding in the ARDS establishment after bacterial infection. Our research was the first to evaluate the EG shedding in the context of ARDS installation after viral infection.

Scientific evidence indicates that EG has an essential function in the vascular permeability control, as well as the adhesion and migration of neutrophils; both of these mechanisms play a well-known role in ARDS pathophysiology [15]. Although a small number of individuals who died were evaluated through autopsy, we evidenced a positive correlation between the soluble thrombomodulin levels and the hyaline membrane perimeter, exhibiting a higher vascular permeability. Orwoll et al. [16] observed in 243 children with ARDS that soluble thrombomodulin levels were associated with organ dysfunction and higher mortality. We revealed a positive correlation between the hyaluronan levels and the number of neutrophils in the alveolar septum, with a higher adhesion and migration of these inflammatory cells. Experimental evidence indicates that EG shedding causes exposure of adhesion molecules leading to leukocyte adhesion to the endothelial surface [17, 18]. 

The mechanisms responsible for EG damage have not yet been fully understood. Some proposals try to explain the virus-triggered EG shedding. The presence of molecular patterns associated with pathogens (PAMPS) would be able to recognize and activate endogenous sheddases, such as the heparanase [19]. The attachment and degradation of sialic acid residues by the neuraminidase (sialidase) of the influenza virus [20, 21], the direct infection of the endothelial cell by the influenza virus [22], or the release of inflammatory cytokines (TNF-α, IL-1β, and IL-6) that activate metalloproteinase can directly cleave proteoglycans, such as syndecan-1 [23]. Currently, some evidence has emerged from the virus-triggered EG damage in other clinical settings, such as during dengue virus infection [24, 25]. 

Evidence exists of cytokine elevation in ARDS patients, mainly in the bronchoalveolar lavage (TNF-alpha, IL-1β, IL-6, and IL-8), but also in the blood (I-2, IL-4, IL-6, IL-8, and IL-18) [26]. However, we did not observe blood elevation of cytokines in these ARDS patients. The two explanations for this finding are, first, we collected this blood sample early during hospitalization, and the majority of the patients had a short period of symptoms, and the cytokines probably increased later. Second, the small sample size was not sufficient to demonstrate a difference in the blood cytokine levels between the groups.

We confirmed the influenza A infection in 57% of the patients with flu syndrome in the ARDS group and 36% of the patients with the flu syndrome in the group without ARDS. This finding could be justified because we first used direct immunofluorescence for viral identification in the nasopharyngeal swab material, a less sensitive test. Only the positive immunofluorescence cases were submitted to the gold standard technique, which is the real-time polymerase chain reaction (RT-PCR). It is essential to highlight that the patients were very well selected during a period of influenza outbreak and that patients with clinical suspicion of bacterial infection were excluded.

There are some limitations to our investigation. First, the major limitation was that the flu syndrome without ARDS patients might not be appropriately selected. Usually, in clinical practice, most flu syndrome patients showed two extreme presentations: a mild form with only upper airway symptoms or a severe form of respiratory failure and ARDS development. This duality causes a problematic task to pair these flu syndrome patients with the severity of their disease. This circumstance affected the patients’ inclusion in the flu syndrome without the ARDS group, in which only 17% was hospitalized. Second, we did not perform the histological analyses of the EG in the lung parenchyma of these patients. It is a difficult methodological process because EG is an unstable structure. In experimental studies, the researchers need to perfuse the animals rapidly after death to guarantee the EG preservation, but in the human being, that is difficult. Third, we cannot definitely confirm that the increased serum hyaluronan was derived from the pulmonary vessels, because we did not perform the measure of this biomarker in the bronchoalveolar lavage fluid. Fourth, this study was a single-centered cross-sectional study, including a small number of patients and a small number of individuals who underwent autopsy. Fifth, it is difficult to exclude bacterial infection definitely in clinical practice; however, these patients were well selected during the influenza outbreak, and those with some possibility of bacterial infections were ruled out.

## Conclusion

These findings suggest that EG shedding happens during the virus-induced ARDS installation. The hyaluronan is a potential biomarker that could be used during flu outbreaks to select patients with a high risk of complications such as the ARDS development, which requires intensive clinical surveillance. However, further investigation should be designed using hyaluronan in a large number of patients with flu syndrome prospectively followed to observe ARDS development.

## Data Availability

The data used and/or analyzed during the current study are available from the corresponding author on reasonable request.

## Abbreviations

ARDS: Acute respiratory distress syndrome
AUC: Area under the curve
CI: Confidence interval
EG: Endothelial glycocalyx
IQR: Interquartile range
PAMPS: Molecular patterns associated with pathogens
RT-PCR: Real-time polymerase chain reaction
ROC: Receiver Operating Characteristic
RR: Relative risk
WHO: World Health Organization
